# Impact of sialic acids on the molecular dynamic of bi-antennary and tri-antennary glycans

**DOI:** 10.1038/srep35666

**Published:** 2016-10-19

**Authors:** Alexandre Guillot, Manuel Dauchez, Nicolas Belloy, Jessica Jonquet, Laurent Duca, Beatrice Romier, Pascal Maurice, Laurent Debelle, Laurent Martiny, Vincent Durlach, Stephanie Baud, Sebastien Blaise

**Affiliations:** 1 Université de Reims Champagne-Ardenne, UMR CNRS 7369, “Matrice Extracellulaire et Dynamique Cellulaire”, UFR Sciences Exactes et Naturelles, Chemin des Rouliers, 51100 Reims, France; 2 Plateau de Modélisation Moléculare Multi-échelle, UFR Sciences Exactes et Naturelles, Chemin des Rouliers, 51100 Reims, France; 3 Pôle Thoracique-Cardio-Neuro-Vasculaire, Centre hospitalier universitaire, 45 Rue Cognacq-Jay, 51100 Reims, France.

## Abstract

Sialic acids (SA) are monosaccharides that can be located at the terminal position of glycan chains on a wide range of proteins. The post-translational modifications, such as N-glycan chains, are fundamental to protein functions. Indeed, the hydrolysis of SA by specific enzymes such as neuraminidases can lead to drastic modifications of protein behavior. However, the relationship between desialylation of N-glycan chains and possible alterations of receptor function remains unexplored. Thus, the aim of the present study is to establish the impact of SA removal from N-glycan chains on their conformational behavior. We therefore undertook an *in silico* investigation using molecular dynamics to predict the structure of an isolated glycan chain. We performed, for the first time, 3 independent 500 ns simulations on bi-antennary and tri-antennary glycan chains displaying or lacking SA. We show that desialylation alters both the preferential conformation and the flexibility of the glycan chain. This study suggests that the behavior of glycan chains induced by presence or absence of SA may explain the changes in the protein function.

Sialic acids (SA) are electronegatively charged monosaccharides in higher animals and some microorganisms. They contribute to the wide structural diversity of complex carbohydrates, which are major constituents of most proteins and lipids of cell membranes and secreted macromolecules^1^. SA are prominently positioned, usually at the outer end of these molecules. The diversity of glycan chains is even more increased by the biosynthesis of various kinds of SA. In human, the number of SA types is limited, with N-acetylneuraminic acid (Neu5Ac) prevailing and followed by derivatives which are O-acetylated and O-lactylated at the SA side chain^2^. The external position of SA on glycoproteins, either alone or in oligo- or polymeric form, implies a strong influence in cell biology. Indeed, these acidic monosaccharides may easily interact with components at other cell surfaces, extracellular substances and effector molecules. Evidence is increasing that they are involved in a multiplicity of cell signalling events. For instance, we have shown previously that the desialylation of the insulin receptor by the neuraminidase-1 sialidase induces the dysregulation of cell glucose uptake and develops insulin resistance^3^,^4^. Moreover, the desialylation of receptors such as PDGF-R or IGF-R alters cell proliferation^5^. In the literature, several authors suppose that SA function is either to mask recognition sites^6^, or, contrarily, to act as a biological target that allows recognition by a receptor protein^7^,^8^. However, the molecular process which leads to receptor alteration after the removal of SA is still unknown. The multiple combinations of glycan chain and the technical limitation of *in vitro* approaches are a crucial bottleneck to the understanding of SA function. This is even more complicated by the fact that the environment of these monosaccharides and the nature of the molecule to which they are bound may influence their biological effects. The aim of the present study is to characterize by molecular dynamics the behaviour of a single N-glycan chain detached from its protein, associated or not with SA. To our knowledge, this strategy has never been used and allows us to predict the stereotypic behaviour modifications of an isolated desialylated glycan. This work is necessary before any complementary study including a complex protein environment around the glycan.

Molecular dynamics simulation is an effective tool for the study of very labile (*i.e.* displaying numerous degrees of freedom) molecule such as glycans^9^,^10^,^11^. This methodology has been employed since the early ages of glycans molecular modeling, twenty-five years ago. At this time, technical limitations allowed scientists to perform simulations for a few hundreds of picoseconds, but this small amount of time was sufficient to predict some glycan conformers with success^12^. In the present study, we performed similar simulations but the sampling was greatly improved as our simulations are three thousand time longer: for each chain the molecular dynamics simulations were performed in order to reach a total amount of 1.5 μs. We used both bi-antennary and tri-antennary chains, thus allowing us to work with sialylated structures commonly found on glycoproteins and for which many data are already available in literature. With those simulations, we predicted the modifications caused by the removal of SA on the glycan chain. We show that the desialylation process alters the preferential conformational state and modifies dihedral angles distribution. We also show with a singular visualization method we propose, the “umbrella visualization”, that those changes lead to the modification of the covered area provided by the glycan on a potential protein. Therefore, these results are the first step to understand the intrinsic properties of glycan chains before analysing the sialidase effects on the glycoproteins.

## Results

### Sialylated and non-sialylated bi-antennary chains display different conformations

**Figure 1:**
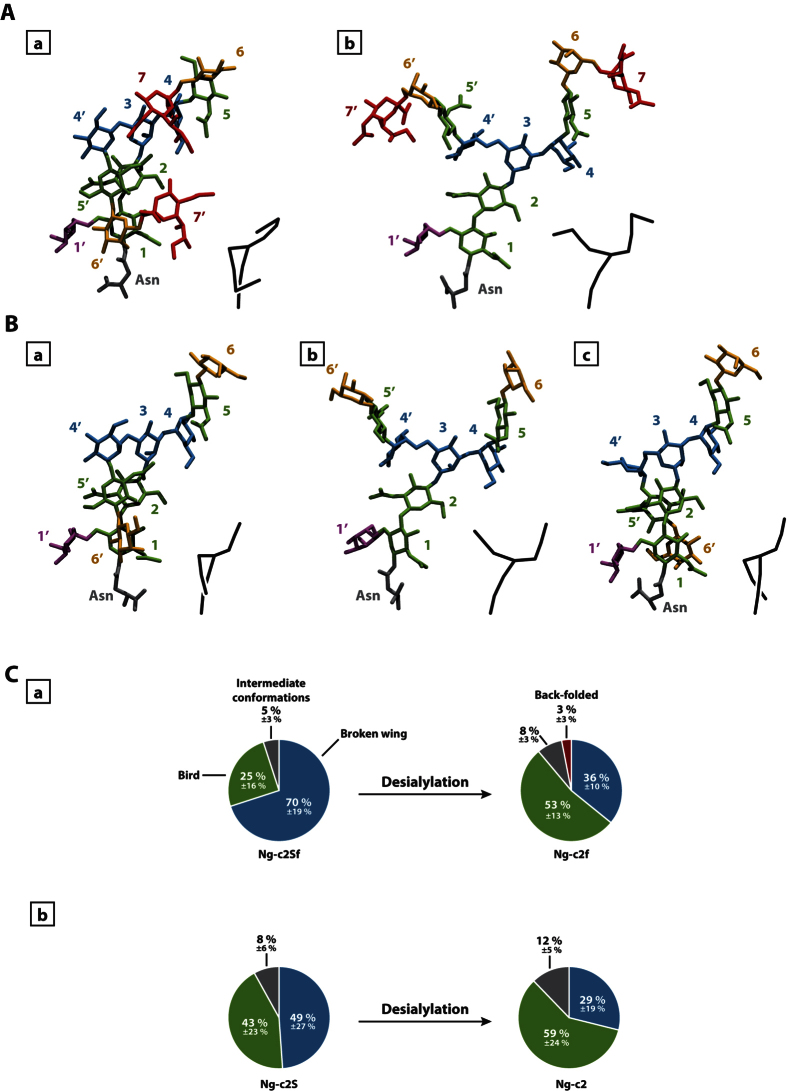
Identification of conformational families. Representation of the most common –conformational states of monofucosylated disialylated bi-antennary glycan (Ng-c2Sf) (**A**) and monofucosylated bi-antennary glycan (Ng-c2f) (**B**). “Broken-wing” (a), “Bird” (b), and “back-folded”(c) conformations. The numbers refer to the numbering used in Fig. 4 and the color-codes are as follows: sialic acids in red, galactose in orange, mannose in blue, N-acetylglucosamine in green, fucose in purple, asparagine in grey. A schematic representation of the chain is provided at the bottom right of each visualization. (**C**) Distribution in percentage of each confor-mational state for Ng-c2Sf (a) and Ng-c2S (b) before and after the removal of sialic acids (“Broken wing” in blue, “bird” in green, “back-folded” in red, and intermediate conformations in grey).

In order to identify the main conformations adopted by sialylated and non-sialylated glycan chains, we performed a clustering analysis on the simulations. For the bi-antennary glycans, the main clusters allowed us to identify some of the conformational states described in previous studies^12^. Among those conformations described at the time, we mainly found the “broken wing” (α1-6 antenna along the inner-core, Fig. 1Aa), and the “bird” (α1-6 antenna perpendicular to the inner-core, Fig. 1Ab). However, the conformational state proportions varied for each glycan chain (Fig. 1C). Indeed, for the disialylated monofucosylated bi-antennary glycan (Ng-c2Sf), the “broken wing” conformation was observed during 70% of the 3 simulations, and the “bird” conformation for 25% of the simulations. The 5% remaining corresponded to intermediate or other conformational states.

**Figure 2:**
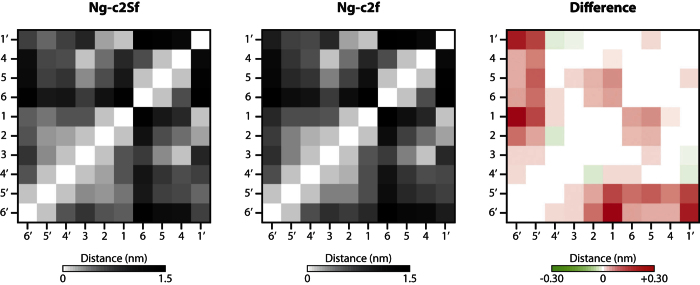
Desialylation process causes an opening of the glycan chain. Contact map of each block of the glycan (smallest average distance between each block). The third map corresponds to the differences between the two first maps (Ng-c2f minus Ng-c2Sf): red means a growing gap, green means a rapprochement after desialylation.

Without SA, the “broken wing” conformation was decreased from 70% to 36%. In parallel, the “bird” conformation increased from 25% to 53%. Moreover, a third conformation also described in the past emerged: this one is named the “back-folded” conformation (α1-6 antenna folded behind the inner-core. Fig. 1Bc). This conformational state represented only 3% of the simulation. The same experiment was also performed on disialylated bi-antennary glycan (Ng-c2S). Here, the removal of SA highly decreased the “broken wing” conformation from 49% to 29% and the “bird” conformation was increased from 43% to 59% (Fig. 1Cb). These data suggest that SA influence the distribution of each conformational states during the simulation. This hypothesis was supported by the analysis of the contact map of both sialylated and non-sialylated chains (Fig. 2). The Gromacs g_mdmat tool was used to visualize the mean distance between each block (each residues). The comparison between maps with or without SA allowed us to estimate the growing gap between each block following the desialylation process. Thus, we show that the desialylation of the glycan chain increases the distance between each block.

**Figure 3:**
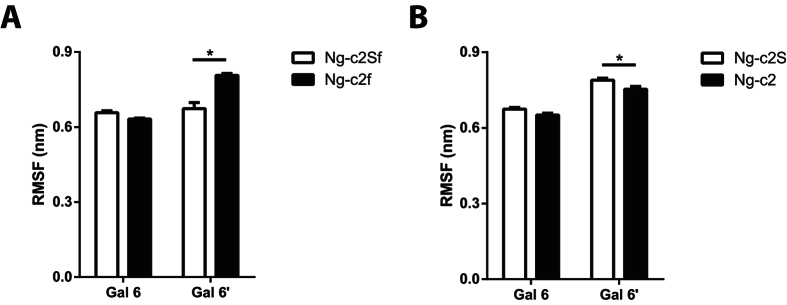
Fluctuation of each antenna increases after desialylation process. Root mean square fluctuation (RMSF) of galactose of each antenna (Gal6 and Gal6′) before and after removing sialic acid from a (**A**) disialylated monofucosylated bi-antennary glycan (Ng-c2Sf) and (**B**) disialylated bi-antennary glycan (Ng-c2S). Statistical test: *p < 0.05.

Interestingly, when SA were removed from the bi-antennary chains, the clustering process was more difficult as the number of clusters was increased for the same cut-off value (0.3 nm). Indeed, the Ng-c2Sf counted an average of 20 clusters. The suppression of SA (Ng-c2f) carried this number up to 32 clusters (from 26 to 38 clusters for Ng-c2S and Ng-c2). In the same way, the number of intermediate structures increased with the non-sialylated form: from 5% of intermediate structures to 9%. In parallel we measured the root mean square fluctuation (RMSF) of galactoses as an indicator of the antenna mobility (Fig. 3). We measured a RMSF value of 0.66 and 0.67 nm for both antennas with SA. In the absence of SA, the RMSF was increased for the α1-6 antenna from 0.67 to 0.81 nm (Fig. 3A). The removal of SA did not significantly change the RMSF value of α1-3 antenna but it slightly decreased on the α1-6 antenna on the Ng-c2S chain (from 0.78 to 0.75 nm, Fig. 3B). Finally, we estimated the number of transitions between the “bird” and the “broken wing” conformation during the 3 simulations. We counted 16 transitions for Ng-c2ASf and 33 for Ng-c2AS. When SA were removed, the number of transitions increased to 47 and 61 transitions for Ng-c2f and Ng-c2, respectively. Those results suggest that while glycans can adopt preferential conformation states, they remain flexible and mobile structures. Moreover, SA seem to be able to influence both of these two aspects of the glycan, independently of the presence or the absence of fucose.

### Dihedral angles change after desialylation process of bi-antennary chains

**Figure 4:**
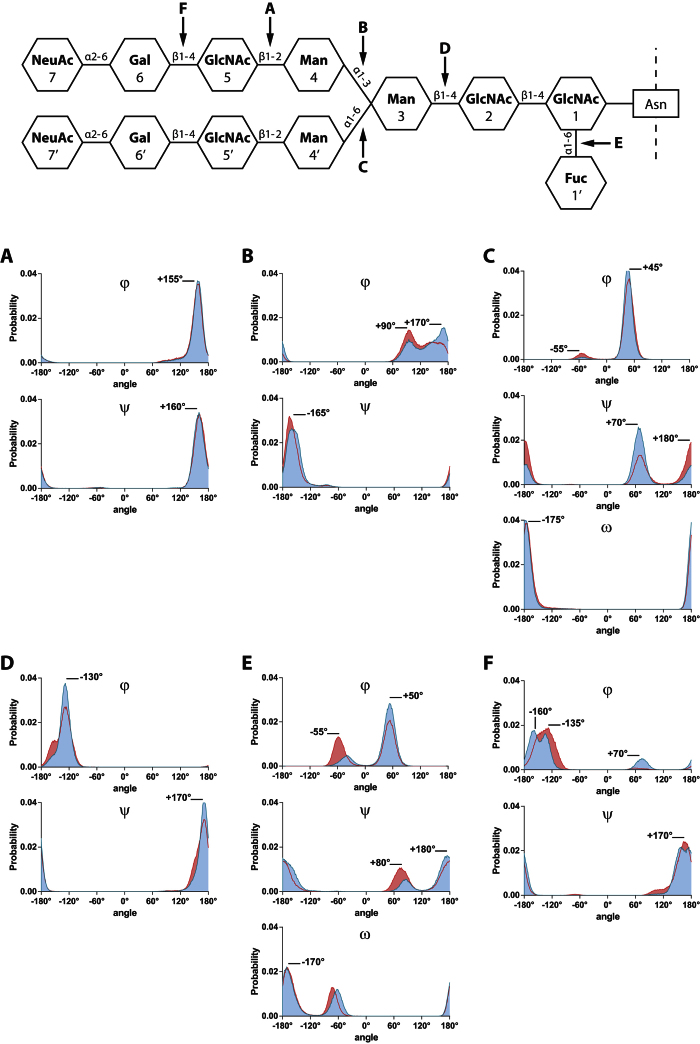
Removing sialic acids does not modify dihedral angles distribution in an equal way. Distribution of dihedral angles (in degree) during MD simulations of the disialylated monofucosylated bi-antennary glycan (Ng-c2Sf, blue lines and surfaces) and the non-sialylated glycan (red lines and surfaces). (**A**) GlcNAc5(β1-2)Man4, (**B**) Man4(α1-3)Man3, (**C**) Man4′(α1-6)Man3, (**D**) Man3(β1-4)GlcNAc2, (**E**) Fuc1′(α1-6)GlcNAc1, (**F**) Gal6(β1-4)GlcNAc5.

As described^13^,^14^, the measurement of dihedral angles involved in glycosidic linkages provides a good evaluation of glycan chain’s flexibility. Thus, we measured the φ and ψ (1–2; 1–3; and 1–4 linkages), or φ, ψ and ω (1–6; and 2–6 linkages) dihedral angles for each glycosidic bond present in the chain (Table S1 and Fig. 4 blue lines).

Although each angle displays different value, we can characterize those dihedral angles by their distribution profile. On Ng-c2Sf, the GlcNAc5(β1-2)Man4 glycosidic bond displayed an unimodal distribution around +160° for both φ and ψ angles (Fig. 4A), suggesting that this glycosidic bond is not an important flexibility point of the chain and remained particularly stable during the whole simulation. On the contrary, the φ Man4(α1-3)Man3 angle showed 2 major peaks at +90° and +170°, but was able take a large range of values from +60° to +180° (Fig. 4B). The ψ Man4′(α1-6)Man3 angle displayed a bimodal distribution, with two angles explored: +70° for 79% of the simulation and +180° for the 21% remaining (Fig. 4C). Those linkages exhibited an increased flexibility, either by having a large range of explored angles, or by displaying a plurimodal distribution.

As described in Table S1 and Fig. 4, in non-sialylated glycan configuration (red lines and surfaces), the glycosidic linkages belonging to the inner-core (blocks 1, 2, and 3, e.g. Fig. 4D) remained almost identical after the removal of SA. In contrast, the desialylation process caused some modifications on Fuc1′(α1-6)GlcNAc1 and Gal6(β1-4)GlcNAc5 angles (Fig. 4E,F). Nevertheless, the position of those angles on the chain (mostly at the end part) limited the impact of their changes towards the overall glycan conformation. The strongest variations were observed on the φ Man4(α1-3)Man3 dihedral angle: the distribution of the two peaks at +90° (35%) and +170° (65%) was inverted so that the +90° angle represents 52% of the simulation, and the +170° angle represents the 48% remaining (Fig. 4B). In the same way, ψ angle from the Man4′(α1-6)Man3 glycosidic linkage displayed bimodal distribution and saw the respective representativeness of each peak modified after the removal of SA (Fig. 4C). The same experiment was performed on the Ng-c2S and returned comparable results on antennas flexibility. However, the absence of the fucose seemed to allow more motions on the inner-core in comparison with Ng-c2Sf as the angles displayed a greater displacement or distribution modification. Taken together, these results suggest that the desialylation process is more likely to modify dihedral angles belonging to the antennas or being involved in the antenna linkage to the inner-core, such as Man4(α1-3)Man3 or Man4′(α1-6)Man3 glycosidic bonds.

### New visualization of the mobility of bi-antennary chains: “umbrella visualization”

To correlate the changes observed on the non-sialylated glycan chain with a potential impact on the protein accessibility, we developed a new method to visualize our results: we called this representation the “umbrella visualization”. As described in the materials and methods section, this representation allowed us to estimate the protein surface that would be shadowed or covered by each antenna of the glycan chain.

**Figure 5:**
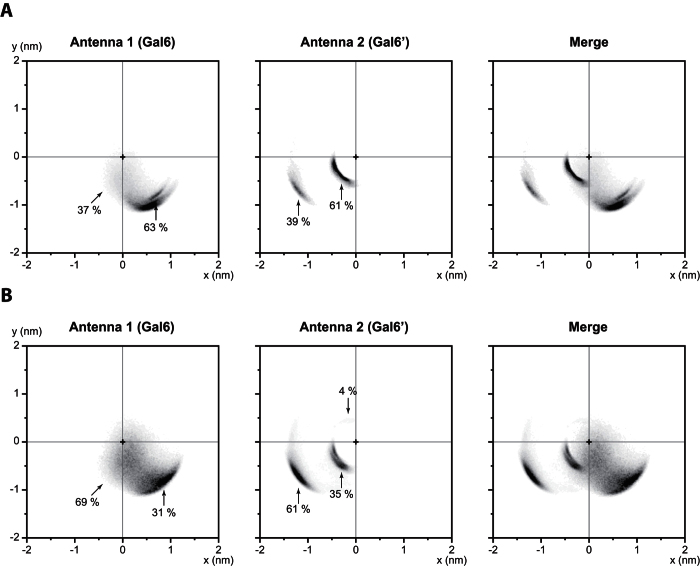
Desialylation process alters the covered area of each antenna. “Umbrella visualization” of both antennas for disialylated monofucosylated bi-antennary glycan (Ng-c2Sf) (**A**), and monofucosylated bi-antennary glycan (Ng-c2f) (**B**).

On disialylated monofucosylated bi-antennary glycan (Fig. 5A), the first antenna mainly covered one spot located at 1.06 nm from the center of the inner-core (GlcNAc1; GlcNAc2; Man3) at coordinates (0.7, −0.8). This position was covered in 63% of the simulation, the 37% remaining were spread between the (0.7, −0.8) spot and the inner-core. Meanwhile, the second antenna was able to cover 2 very distinct positions. The first one was the most important as it represented 61% of the total simulation (−0.4, −0.3) and was located very close from the inner-core of the glycan chain, at 0.50 nm. On the contrary, the second spot was located far from the inner-core (−1.2, −0.7), at 1.39 nm and represented 39% of the simulation.

After the removal of SA (Fig. 5B), on the first antenna, the spot located at (0.7, −0.8) was still present. Though, it became less representative with only 31% of the simulation (63% when SA are present). Moreover, the others positions (69%) were widely spread from the original position to the inner-core in a square of 1.5 nm side. The second antenna also displayed changes in its distribution. The two spots previously described were still present but their respective representativeness changed. The first one was located close from the inner-core (−0.4, −0.3) and its proportion decreased from 61% to 35% after desialylation. At the same time, the second spot, far from the inner-core (−1.2, −0.7), increased from 39% to 61% when SA are removed. Finally, a third spot appeared next to the inner-core (0.50 nm), at coordinates (−0.3, 0.4). This spot represented only 4% of the total simulation. In parallel, we showed that the distance between the end of each antenna and the inner-core could be correlated with only a few specific dihedral angles. Indeed, the α1-6 antenna profile could be associated with the ψ Man4′(α1-6)Man3 dihedral angle with a Spearman’s rank correlation coefficient of 0.60 (Ng-c2Sf) and 0.76 (Ng-c2f). And the α1-3 antenna profile was correlated with the φ Man4(α1-3)Man3 dihedral angle with a coefficient of 0.84 (Ng-c2Sf) and 0.87 (Ng-c2f). When merged, those results show that removing SA increases the explored area by both antennas of the glycan chain. Moreover, only a few glycosidic bonds seem to be involved in this process. This suggest that the removal of SA from the glycan chain causes modification of the coverage profile and could impact the protein accessibility.

### Sialylated and non-sialylated tri-antennary glycan chains display different conformations

**Figure 6:**
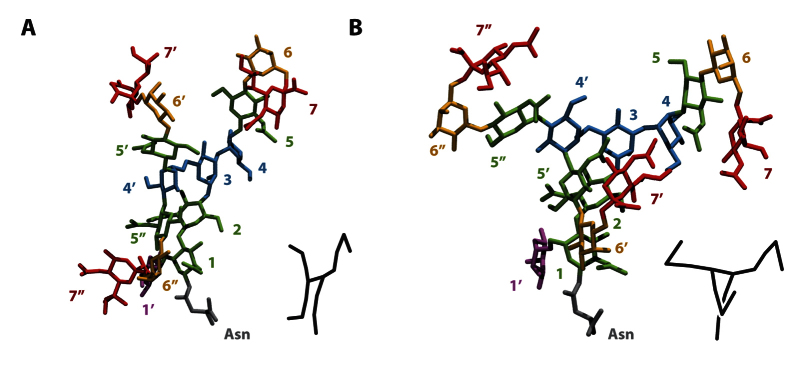
Main conformational families of tri-antennary glycan. Representation of the most common conformational states of monofucosylated trisialylated tri-antennary glycan (Ng-c3Sf). GlcNAc5″, Gal6″, and NeuAc7″ antenna is folded along the inner-core (**A**); GlcNAc5′, Gal6′, and NeuAc7′, antenna is folded along the inner-core (**B**). The numbers refer to the numbering used in Fig. 7.

Among the important varieties of existing N-glycan, the bi-antennary model is the most studied^12^,^15^. However, N-glycan can also be present in a tri-antennary form at the surface of human protein^16^,^17^. As a consequence, we decided to extend our study to the trisialylated monofucosylated tri-antennary glycan (Ng-c3Sf). The clustering process applied on this structure allowed us to find one major conformational family representing 68% of the simulation (Fig. 6A). On this conformation, the new added antenna (GlcNAc5″, Gal6″, and NeuAc7″) folded along the inner-core, reminding the “broken wing” conformational state previously described on bi-antennary chains ((Fig. 1Bc). Interestingly, a rotation around the Man4′(α1-6)Man3 linkage was also able to invert the position of the second (GlcNAc5′, Gal6′) and the third antenna (11% of the simulation Fig. 6B). The position of the glycan was then highly stackable with the bi-antennary equivalent conformation. When SA was removed, this predominant conformational state became even more representative of the simulation (87%). This stabilization of the glycan conformation was also confirm by the measurement of the RMSF. The removal of SA decreased the RMSF from 0.60 nm to 0.53 nm (2^nd^ antenna, Gal6′) and from 0.84 nm to 0.57 nm (3^rd^ antenna, Gal6″). The absence or the presence of the fucose on the inner-core did not deeply modify the global arrangement of the glycan. However, this residue limited the rotation around the Man4′(α1-6)Man3 by blocking the end of the second and the third antennas. Those results suggest that, despite the addition of a new antenna, similar structures can be found between bi- and tri-antennary glycan chains. Moreover, as observed on bi-antennary glycan, the removal of SA does impact the global arrangement of those structures.

### Dihedral angles change after desialylation process of tri-antennary chains

**Figure 7:**
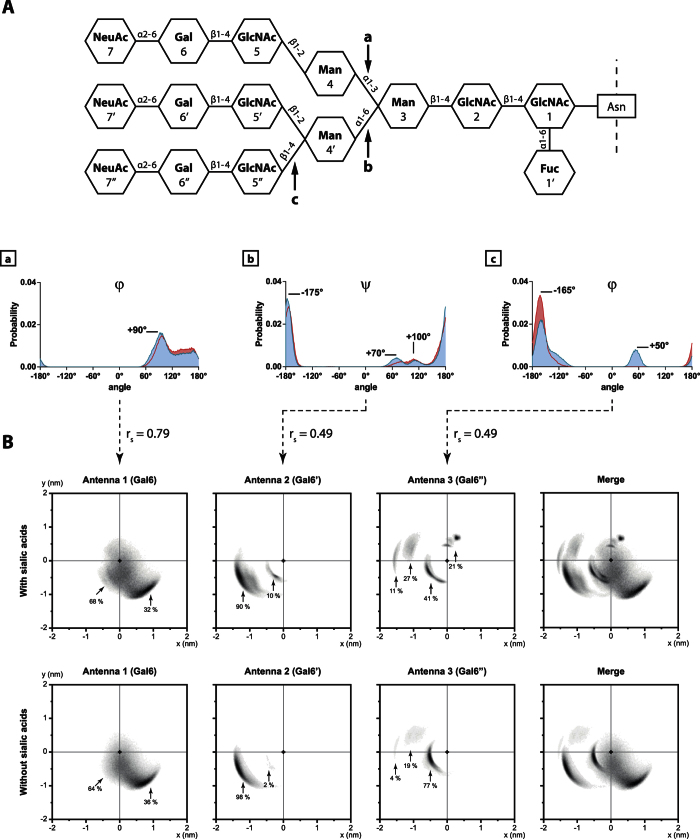
Desialylation process modifies dihedral angle distribution and the hypothetic protein covered surface by tri-antennary glycans. (**A**) Distribution of dihedral angles (in degree) involved in antennas linkage: (a) Man4(α1-3)Man3, (b) Man4′(α1-6)Man3, (c) GlcNAc5″(β1-4)Man4′. With sialic acids (blue lines and surfaces) and without (red lines and surfaces). Some of those angle distributions display a significative Spearman correlation coefficient (r_s_) with the “umbrella visualization” profile (**B**) of tri-antennary glycans.

The measurement of dihedral angles provided similar results as the ones obtained on bi-antennary glycans. Most of the structure flexibility was caused by few glycosidic bonds involved in antennas linkage: Man4(α1-3)Man3, Man4′(α1-6)Man3, or GlcNAc5″(β1-4)Man4′ (Fig. 7A and Table S2). Furthermore, when SA were removed, those angles showed some variations in their distribution. The φ Man4(α1-3)Man3 dihedral angle was distributed on one large peak at +90° and is then spread between +90 and +170°. After the removal of sialic acid 20% of the +90° peak is displaced towards +170° (Fig. 7Aa). The φ GlcNAc5″(β1-4)Man4′ angle, involved in the linkage of the third antenna, passed from a bimodal distribution (+50° and −165°) to an unimodal (−165°) (Fig. 7Ac). Obviously, those changes in dihedral angles impact the way the glycan move, either with, or without SA. Moreover, as observed on bi-antennary glycans, there is a correlation between those specific dihedral angles, and the “umbrella visualization” profile of their associated antennas.

### “Umbrella visualization” of tri-antennary chains

The “umbrella visualization” of the first antenna gave a similar profile to the one characterizing the bi-antennary chain. This antenna explored a wide area from 0 to around 1 nm distance from the inner-core, regardless of the presence or the absence of SA (Fig. 7B). The second antenna explored the same spot located at (−1.3, −0.5) for most of the simulation (90%). This antenna was also able to explore another area closer from the inner-core, at (−0.3, −0.4) for about 10% of the simulation. Finally, the third antenna was located at various positions around the inner core. Two of these positions correspond to short distances from the origin: at coordinates (0.3, 0.7) for 21% and at coordinates (−0.4, −0.3) for 41% of the simulation. The two other spots are situated further from the inner-core and correspond to 27 and 11% of the simulation. When SA were removed, this antenna mostly explored (77% of the simulation) the spot located at (−0.4, −0.3). According to those results, it appears that the desialylation process impacts the tri-antennary glycan chain in a different way than the bi-antennary chain. Here, only the third antenna seems to be deeply impacted by the removal of SA.

## Discussion

Many studies have examined the roles of N-glycosylation on the stability and on the structure of proteins^18^,^19^. Some of these N-glycosylation chains exhibit sialic acids on their terminal portion that may be cleaved by sialidases, also named neuraminidases, thus leading to the disruption of the functionality of the protein^3^,^5^. Indeed, SA are acidic monosaccharides typically found at the outermost ends of the sugar chains of animal glycoconjugates. Even though they are involved in the intermolecular and intercellular interactions, they also act as critical components of ligands recognized by a variety of proteins of animal, plant, and microbial origin (sialic acid binding lectins)^20^,^21^,^22^. Recognition can be affected by: specific structural variations and modifications of SA, their linkage to the underlying sugar chain, the structure of these chains, and the nature of the glycoconjugate to which they are attached. The biological studies show that the desialylation induced by neuraminidases, alters the function of glycoproteins. In this study, we show for the first time with molecular dynamics simulations the structural consequences of the desialylation of N-glycan chains. To achieve this goal, we performed 1.5 µs extensive simulations at 310 K. This important sampling was long enough to analyze both the flexibility of the glycan with or without SA, and its capacity to adopt preferential conformations.

In presence of SA, bi-antennary chains can be classified either in the “back-folded”, “bird”, or “broken wing” group, this last being the most representative of each simulation, according to Mazurier J *et al.*^23^. While the global arrangement of common blocks is not changed, the desialylation process modifies the representativeness of each arrangement. The “broken wing” conformation becomes less representative to the profit of the “bird” or of the back-folded conformation. In the “broken wing” conformational state, the interaction between the two SA is able to lock and stabilize the arrangement. Thus, by removing SA, we allow the chain to open more widely the antennas (*e.g*. “bird” conformation). This “opening” process is also visible with the contact map in which the removal of SA increases the gap between blocks from each other’s (Fig. 2) and reflects the fact that SA interact strongly with the “trunk” of the glycan (constituted by the following blocks: GlcNAc 1 - GlcNAc 2 - Man 3). Indeed, we observe shorter distances between the GlcNac 1 block and the Gal 6′ block upon presence of the sialic acids. Given the nature of the sialic acid, these interactions are mainly stabilized through hydrogen bonds which tend to fold one arm of the umbrella against the “trunk”. The second consequence of this interaction is that the “folded arm” loses its flexibility: this is directly observable through the decrease of the Gal 6′ RMSF upon sialylation. Moreover, in bi-antennary chain without SA, the clustering process is more difficult than in N-glycan chain with SA. The intermediate structures obtained and the measurement of RMSF are two other arguments of the SA role in the stability of N-glycan chains.

The mobility and the ability of the glycan to adopt a particular conformation mostly depends on the configuration of the glycosidic linkages between each block^13^. The cyclic structure of the sugars reduces the degree of freedom of each chain components. The measurement of dihedral angles involved in glycosidic linkage remains one of the best way to evaluate the flexibility of the chain point by point. In agreement with Dauchez *et al.*, we also show that all glycosidic linkages are not equally flexible^15^.

N-glycans are post-translational structures essential for the functionality of the associated protein, protein-protein interactions or cell-cell interactions. This underlines the importance to be able to visualize the protein covered area. The cluster identifications provide an easy way to observe glycan arrangements, but they only give a static analysis of the whole simulation and do not allow to appreciate the dynamic aspect on the investigated glycan chains. Conversely, the measurements of dihedral angles and RMSF provide a better description of the different motions, but the visualization of the results is less intuitive and remains difficult. Thus, we decided to consider the glycans as an opened umbrella where the antennas, with or without SA, are the whales. Indeed, such a structure should prevent the interaction between the protein of interest and other partners. As an example, the modifications of the electrostatic properties of the protein or the steric hindrance could prevent the protein from interacting with unwanted partners such as proteases or pathogens^24^,^25^. Therefore, we present, for the first time, a new representation of glycan chains taking into account both, the main positions adopted by each antenna of the glycan and the intensity of their motions. The “umbrella visualization” is based on the shade of the glycan chains projected on a plan, thus mimicking the protein shadowed surface by the antennas. This system allows us to discuss about both the flexibility of the glycan (i.e. its ability to explore very distinct areas), and the stability of the glycan (i.e. its ability to avoid spreading from the main conformational state). Indeed, the visualization of overlapping areas on the plan shows that among the two antennas, the α1-6 antenna is more flexible because it can explore several distinct conformational states. But this antenna is also more stable as it does not spread far from these positions. Conversely, as observed with the “umbrella visualization”, the α1-3 antenna can only take one average position, but stays in motion during the simulation and explore the space around this position. The “umbrella visualization” also allows us to corroborate the results obtained with the clustering process and with the dihedral angle measurement. Indeed, the profile obtained with the α1-6 antenna displays 2 spots located either close or far from the inner-core. Both those locations can be correlated with the two main clusters obtained in Fig. 1. The nearest spot corresponds to the “broken wing” conformational state, where the antenna is folded along the inner-core, and the second spot finds its origins with the “bird” conformations where the antenna is moved far from the inner-core. Finally, the desialylation process causes the emergence of a third spot near the inner-core, which corresponds to the “back-folded” conformation. We were also able to establish correlations between the distance of the antenna’s end from the inner-core and dihedral angle measurement. Interestingly, we show that the motion of the antenna can be correlated with only a few dihedral angle. The α1-6 antenna profile can be associated with the ψ Man4′(α1-6)Man3 dihedral angle and the α1-3 antenna profile is correlated with the φ Man4(α1-3)Man3 dihedral angle. This set of results shows that the removal of SA from the bi-antennary glycan chain is likely to notoriously modify the interaction between the glycan and the protein and the covered surface.

A large number of glycan structures has already been identified. They have been divided in three groups: complex, mannose and hybrid type. For each group, the glycan will vary in length, composition, and number of antennas. From the past decades, the monofucosylated disialylated bi-antennary glycan has been the most studied type of glycosylation^26^. Nevertheless, several proteins such as human immunoglobulin G present the fucosylated tri-antennary glycan form^27^,^28^. To our knowledge, our work is the first to report the role of SA on tri-antennary glycan using extensive molecular dynamics simulation (1.5 μs). Interestingly, the clustering process shows that, with SA, the conformations and dihedral angles adopted by tri-antennary glycans are similar to those presented by bi-antennary glycans. The slight differences observed might be due to the glycosidic bonds involved in antennas linkage. In contrast, the similarity observed between bi-antennary and tri-antennary chains associated with SA, is lost when SA are removed. On the bi-antennary glycan, the desialylation process causes an “opening” of the structure by promoting the “bird” conformation instead of the “broken wing” conformation, and increase its mobility. Meanwhile, the removal of SA from tri-antennary glycan generates a change in the average conformation of the new added antenna and decreases its mobility.

In conclusion, we show for the first time that the removal of SA from the terminal position of each antenna can lead to various modifications of the glycan behavior, depending on the studied model. Nevertheless, the final consequences remain identical in both bi-antennary and tri-antennary glycans: the covered area provided by the glycan chain on a hypothetical protein surface is modified. As mentioned previously, the changes observed in the structural and dynamical behavior of the glycan chains upon desialylation originate from the loss of interactions between the trunk and one of the arm, thus releasing it. Although, we focused this study on the impact of the desialylation on isolated glycan chains, we believe that it is the first step towards the understanding of the influence of SA on the structure and functions of proteins at the atomic and molecular level. Indeed, the desialylation process leads to the modification of the protected surface of the protein, and thus the protein/glycan interaction. The stability of N-glycan chain structure could be an important element to take in account in the immobilization process of proteins. For example, at the surface of sero-transferrines, the glycan chains present a “broken wing” arrangement: this conformation reinforces the association of the two lobes and contributes to maintain the protein moieties in a biologically active 3D conformation^29^. Nevertheless, no data show the consequence of sialic acid presence on this protein structure. Thus, the mobility of the N-glycan observed without SA could destabilize the structure of the protein or the interaction with other partners and explain why several proteins without SA exhibit an inhibition of their functions such as EGFR and IGFR^5^,^30^.

## Methods

### Starting structure

All structures are built using the Avogadro software. Each block of the glycan chain is separately built and submitted to energy minimization steps. The chain is then assembled block by block with energy minimization at each step until the full glycan structure is obtained. Modified version of the OPLS-AA force field is used to describe the atoms^31^,^32^. This version has been adapted for our study and describes all the atoms used in our glycan chains models (Table S3). The list of structures and their respective abbreviations is summarized in Table 1.

### Simulation

Molecular dynamic simulations are performed at ROMEO HPC Center, using the GROMACS package 4.6.3^33^. Prior to simulations, each system is submitted to multiple preparation steps. The systems are first minimized in vacuum by 2,500 steps to remove eventual steric clashes (steepest descent energy minimization). Periodic boundary conditions are then applied by generating a cubic box around the structures. This box is then filled with TIP3P explicit water model^34^,^35^, followed by 2,500 steps of energy minimization in solvent. Finally, 500 ps of an NPT molecular dynamics equilibration are performed to bring the system to the target temperature and pressure of 310 K and 1 bar, respectively.

Table S4 summarizes the parameters used for NPT molecular dynamics simulations. 3 independent simulations of 500 ns with different starting points were performed for each system, leading to the total calculation time of 1.5 μs with a 2 fs integration time step^36^,^37^. No counter ions or excess salt were needed. Atomic coordinates are recorded every picosecond and the LINCS algorithm^38^ is used to constrain the bonds with a hydrogen atom. Each system is simulated at the physiological temperature of 310 K.

### Trajectory analysis

Analysis are performed on trajectory files with a temporal resolution of 10 picoseconds.

#### Clustering

The GROMACS g_cluster tool is used to determine most representative conformational states of the glycan chain. Hydrogen atoms are ignored and the clustering gromos method is used during the analysis. We choose the smallest cut-off allowing to classify 90% of the simulation in the first 5 clusters for the sialylated glycan chain.

#### RMSF

The average position of each atom during each 500 ns simulation is calculated to generate the reference structure needed for a Root Mean Square Fluctuation computation. As the galactose is the last common block for both sialylated and non-sialylated antennas, we measure the RMSF from its center of mass as an indicator of antennae’s mobility. To perform a statistical analysis, the global RMSF is calculated using the block averaging method with windows of 10 ns. Thus, 150 RMSF values are generated for each structure. In order to compare sialylated and non-sialylated chains, a *t*-test is performed with a statistical significance (*p < 0.05).

#### Contact map

The GROMACS g_mdmat tool is used to generate contact map between each block of the glycan (smallest average distance between 2 residues). One map is generated for the complete glycan chain, and another one is created for the glycan chain lacking sialic acids. Finally, a third map is created by subtracting the second map to the first map in order to evaluate the growing gap or the rapprochement of each block.

#### Dihedral angle

The GROMACS g_angle tool is used to measure dihedral angles between blocks. φ, ψ, and ω angles are measured between each blocks.

#### “Umbrella visualization”

With the aim to appreciate the covered zone explored by glycan chains on a hypothetic protein, we project the position of each antennae on an oriented xy plan (Supplementary Figure S1). The glycan chain is placed on a xyz coordinate system in such a way that the asparagine residue is set on the origin. With an in house program, we calculate the angle between the z axis and the vector given by the inner-core (blocks 1, 2 and 3: GlcNAc, GlcNAc, Man) of the glycan chain, so the inner-core can be oriented along the z axis. The chain is then oriented around the z axis to keep each antennae on a defined side: the angle between the x axis and the vector given by blocks 4, 3 and 4′ (Man, Man, Man) is calculated and the chain is rotated so that this vector becomes coplanar with the xz plan. Finally, the xy positions of the last common blocks (Gal) for both sialylated and non-sialylated antennas are reported on a new graph.

### Statistical analysis

The Spearman’s rank correlation coefficient is used to estimate correlations between the antennas distance from the inner-core and each dihedral angles of glycosidic linkages. At each one of the 150,000 time steps of the simulation, the distance between the end of the antenna and the inner-core, and the value of the dihedral angles of a glycosidic linkage are read and reported in a table. The Spearman’s rank correlation coefficient is then calculated with p < 0.001.

### Visualization

All visualizations are produced using Visual Molecular Dynamics (VMD)^39^ with tachyon rendering mode^40^.

## Additional Information

**How to cite this article**: Guillot, A. *et al.* Impact of sialic acids on the molecular dynamic of bi-antennary and tri-antennary glycans. *Sci. Rep.* **6**, 35666; doi: 10.1038/srep35666 (2016).

## Supplementary Material

Supplementary Information

## Figures and Tables

**Table 1:** List of abbreviations used for identifying the glycan chains.

	Fucosylation	Sialylation state	Abbreviation
Bi-antennary	Monofucosylated	Disialylated	**Ng-c2Sf**
		Non-sialylated	**Ng-c2f**
	Non-fucosylated	Disialylated	**Ng-c2S**
		Non-sialylated	**Ng-c2**
Tri-antennary	Monofucosylated	Trisialylated	**Ng-c3Sf**
		Non-sialylated	**Ng-c3f**
	Non-fucosylated	Trisialylated	**Ng-c3S**
		Non-sialylated	**Ng-c3**
