# Supplementary material for: Impact of sialic acids on the molecular dynamic of bi-antennary and tri-antennary glycans
Source: Sci Rep. 2016 Oct 19;6:35666. doi: 10.1038/srep35666 (PMC5069492; doi:10.1038/srep35666)
Supplement: Supplementary Information [file srep35666-s1.doc]

**Impact of sialic acids on the molecular dynamic of bi-antennary and tri-antennary glycans**

**Alexandre GUILLOT^1*^, Manuel DAUCHEZ^1,2^, Nicolas BELLOY^1,2^, Jessica JONQUET^1,2^, Laurent DUCA^1^, Béatrice ROMIER^1^, Pascal MAURICE^1^, Laurent DEBELLE^1^, Laurent MARTINY^1^, Vincent DURLACH^1,3,#^, Stéphanie BAUD^1,2^*^,#^, Sébastien BLAISE^1^,*^,#^**

| **Glycosidic bond** | | | **Angle type** | **Distribution**  **(deg)** | **W/ SA**  **(%)** | **W/O SA**  **(%)** |
| --- | --- | --- | --- | --- | --- | --- |
| Fuc1' | α1-6 | GlcNAc1 | ϕ | +50 | 87 | 63 |
|  |  |  |  | -50 | 13 | 37 |
|  |  |  | ψ | +180 | 88 | 62 |
|  |  |  |  | +85 | 12 | 38 |
|  |  |  | ω | -170 | 70 | 70 |
|  |  |  |  | -70 | 30 | 30 |
| GlcNAc2 | β1-4 | GlcNAc1 | ϕ | -160 | 70 | 56 |
|  |  |  |  | -135 | 30 | 44 |
|  |  |  | ψ | +150 | 100 | 100 |
| Man3 | β1-4 | GlcNAc2 | ϕ | -130 | 88 | 75 |
|  |  |  |  | -160 | 12 | 25 |
|  |  |  | ψ | +170 | 100 | 100 |
| Man4 | α1-3 | Man3 | ϕ | +170 | 65 | 48 |
|  |  |  |  | +90 | 35 | 52 |
|  |  |  | ψ | -160 | 100 | 100 |
| GlcNAc5 | β1-2 | Man4 | ϕ | +155 | 100 | 100 |
|  |  |  | ψ | +160 | 100 | 100 |
| Gal6 | β1-4 | GlcNAc5 | ϕ | +70 | 16 | 4 |
|  |  |  |  | -135 | 40 | 68 |
|  |  |  |  | -165 | 44 | 28 |
|  |  |  | ψ | +160 | 100 | 100 |
| Man4' | α1-6 | Man3 | ϕ | +45 | 97 | 93 |
|  |  |  |  | -55 | 3 | 7 |
|  |  |  | ψ | +65 | 79 | 40 |
|  |  |  |  | +180 | 21 | 60 |
|  |  |  | ω | -175 | 100 | 100 |
| GlcNAc5' | β1-2 | Man4' | ϕ | +155 | 67 | 79 |
|  |  |  |  | +110 | 33 | 21 |
|  |  |  | ψ | +155 | 100 | 100 |
| Gal6' | β1-4 | GlcNAc5' | ϕ | -160 | 61 | 31 |
|  |  |  |  | -140 | 39 | 69 |
|  |  |  | ψ | +160 | 100 | 100 |

**Supplementary Table S1.** Dihedral angle distributions and their modifications following the removal of sialic acids on monofucosylated disialylated bi-antennary glycan (Ng-c2Sf).

| **Glycosidic bond** | | | **Angle type** | **Distribution**  **(deg)** | **W/ SA**  **(%)** | **W/O SA**  **(%)** |
| --- | --- | --- | --- | --- | --- | --- |
| Fuc1' | α1-6 | GlcNAc1 | ϕ | +50 | 81 | 68 |
|  |  |  |  | -45 | 19 | 32 |
|  |  |  | ψ | +170 | 89 | 65 |
|  |  |  |  | +85 | 11 | 35 |
|  |  |  | ω | -170 | 67 | 71 |
|  |  |  |  | -70 | 33 | 29 |
| GlcNAc2 | β1-4 | GlcNAc1 | ϕ | -160 | 56 | 67 |
|  |  |  |  | -140 | 44 | 33 |
|  |  |  | ψ | +150 | 100 | 100 |
| Man3 | β1-4 | GlcNAc2 | ϕ | -160 | 42 | 16 |
|  |  |  |  | -130 | 58 | 84 |
|  |  |  | ψ | +180 | 64 | 90 |
|  |  |  |  | +145 | 36 | 10 |
| Man4 | α1-3 | Man3 | ϕ | +90 | 60 | 48 |
|  |  |  |  | > +120 | 40 | 52 |
|  |  |  | ψ | -170 | 100 | 100 |
| GlcNAc5 | β1-2 | Man4 | ϕ | +160 | 100 | 100 |
|  |  |  | ψ | +160 | 100 | 100 |
| Gal6 | β1-4 | GlcNAc5 | ϕ | -135 | 47 | 62 |
|  |  |  |  | -160 | 53 | 48 |
|  |  |  | ψ | +165 | 95 | 98 |
|  |  |  |  | -72 | 5 | 2 |
| Man4' | α1-6 | Man3 | ϕ | +55 | 90 | 99 |
|  |  |  |  | -30 | 10 | 1 |
|  |  |  | ψ | -175 | 78 | 83 |
|  |  |  |  | +100 | 8 | 10 |
|  |  |  |  | +70 | 14 | 7 |
|  |  |  | ω | -170 | 90 | 87 |
|  |  |  |  | -90 | 10 | 13 |
| GlcNAc5' | β1-2 | Man4' | ϕ | +155 | 100 | 100 |
|  |  |  | ψ | +160 | 100 | 100 |
| Gal6' | β1-4 | GlcNAc5' | ϕ | -135 | 44 | 67 |
|  |  |  |  | -160 | 38 | 30 |
|  |  |  |  | +65 | 18 | 3 |
|  |  |  | ψ | +160 | 95 | 98 |
|  |  |  |  | -70 | 5 | 2 |
| GlcNAc5'' | β1-4 | Man4' | ϕ | +50 | 20 | 0 |
|  |  |  |  | -165 | 80 | 100 |
|  |  |  | ψ | +150 | 100 | 100 |
| Gal6'' | β1-4 | GlcNAc5'' | ϕ | -140 | 39 | 64 |
|  |  |  |  | -160 | 61 | 36 |
|  |  |  | ψ | +160 | 100 | 100 |

**Supplementary Table S2.** Dihedral angle distributions and their modifications following the removal of sialic acids on monofucosylated trisialylated tri-antennary glycan (Ng-c3Sf).

| **Dihedral** | **C_0_ (kJ.mol^-1^)** | **C_1_ (kJ.mol^-1^)** | **C_2_ (kJ.mol^-1^)** | **C_3_ (kJ.mol^-1^)** |
| --- | --- | --- | --- | --- |
| **Sialic acid** | | | | |
| 12-10-14-16 | 9.89307 | -4.71746 | 3.67774 | -8.85335 |
| 16-14-24-39 | 2.92880 | -1.46440 | 0.20920 | -1.67360 |
| 3-2-7-10 | -4.96013 | 6.28646 | 1.30959 | -2.63592 |
| 3-2-7-8 | -0.20920 | -0.62760 | 0.00000 | 0.83680 |
| 7-2-3-5 | 5.31786 | 0.73220 | -2.28446 | -3.76560 |
| 7-2-3-4 | 2.28446 | 0.00000 | -2.28446 | -3.76560 |
| 2-3-5-6 | 26.15000 | -3.13800 | -23012.00000 | 0.00000 |
| 3-2-39-24 | 1.71544 | 2.84512 | 1.04600 | -5.60656 |
| 4-3-2-39 | 2.28446 | 0.00000 | -2.28446 | 0.00000 |
| 5-3-2-39 | 14.43480 | -11.00392 | -3.43088 | 0.00000 |
| **Galactose** | | | | |
| 1-2-5-7 | 9.03534 | -9.03534 | 0.00000 | 0.00000 |
| 3-2-5-7 | 0.97905 | 2.93716 | 0.00000 | -3.91622 |
| **Mannose** | | | | |
| 3-2-18-19 | 0.62760 | 1.88280 | 0.00000 | -2.51040 |
|  |  |  |  |  |
| **N-acetylglucosamine** | | | | |
| 10-9-12-14 | 0.97069 | 2.91206 | 0.00000 | -3.88275 |
| 9-12-14-16 | -4.70700 | 2.92044 | 1.78656 | 0.00000 |
| 11-9-12-14 | 2.92880 | -1.46440 | 0.20920 | -1.67360 |
| **Link between Mannose and N-acetylglucosamine** | | | | |
| 3_MAN_-2_MAN_-18_MAN_-11_GLC_ | 0.97905 | 2.93716 | 0.00000 | -3.91622 |
| 2_MAN_-18_MAN_-11_GLC_-9_GLC_ | 1.71544 | 2.84512 | 1.04600 | -5.60656 |
| 4_MAN_-2_MAN_-18_MAN_-11_GLC_ | -1.15060 | -1.15060 | 0.00000 | 0.00000 |
| **Link between N-acetylglucosamine and the asparagine** | | | | |
| C_b_-C_g_-N_d_-9_GLC_ | 30.28798 | -4.81160 | -25.47638 | 0.00000 |
| O_d_-C_g_-N_d_-9_GLC_ | 25.47638 | 0.00000 | -25.47638 | 0.00000 |
| C_g_-N_d_-9_GLC_-8_GLC_ | -3.13800 | -3.13800 | 6.27600 | 0.00000 |
| C_g_-N_d_-9_GLC_-10_GLC_ | -0.29079 | -0.87237 | 0.00000 | 1.16315 |
| C_g_-N_d_-9_GLC_-12_GLC_ | -4.70700 | 2.92044 | 1.78656 | 0.00000 |
| H_d_-N_d_-9_GLC_-8_GLC_ | -1.26775 | 3.02085 | 1.74473 | -3.49782 |
| N_d_-9_GLC_-8_GLC_-6_GLC_ | -5.23000 | 7.32200 | 6.27600 | -8.36800 |
| N_d_-9_GLC_-12_GLC_-13_GLC_ | 0.97069 | 2.91206 | 0.00000 | -3.88275 |
| N_d_-9_GLC_-12_GLC_-14_GLC_ | 19.59940 | -21.39070 | 4.05011 | -2.25936 |
| N_d_-9_GLC_-12_GLC_-22_GLC_ | 5.48732 | 0.02719 | 0.00000 | -5.51451 |

**Supplementary Table S3.** List of the C_n_ parameters of the Ryckaert-Bellemans functions that were adapted from existing OPLS-AA parameters. The numbering refers to the one presented in supplementary figure S2. The atoms labelled as C_β_, C_γ_, N_d_, O_d_ and H_d_ belong to the N-glycosylated asparagine.

| **Parameter** | **Value** |
| --- | --- |
| Package | GROMACS v 4.6.3 |
| Force field | Adapted OPLS-AA |
| Constraint algorithm for H-bond | LINCS |
| Box shape | Cubic |
| Box side size (nm) | 5 (min) to 6 (max) |
| Water model | TIP3P |
| Water molecule | 4500 (min) to 7800 (max) |
| Duration (ns) | 500 |
| Time step (fs) | 2 |
| Nstlist | 10 |
| Ns_type | Grid |
| Rlist (nm) | 1.5 |
| Rcoulomb (nm) | 1.5 |
| Coulomb type | PME |
| PME order | 4 |
| Rvdw (nm) | 1.5 |
| Temperature coupling | v-rescale |
| Tau-t (ps) | 0.1 |
| Ref-t (K) | 310 |
| Pressure coupling | Berendsen |
| Tau_p (ps) | 4 |
| Ref_p (bar) | 1 |

**Supplementary Table S4.** Molecular dynamics simulations, list of parameters.

**
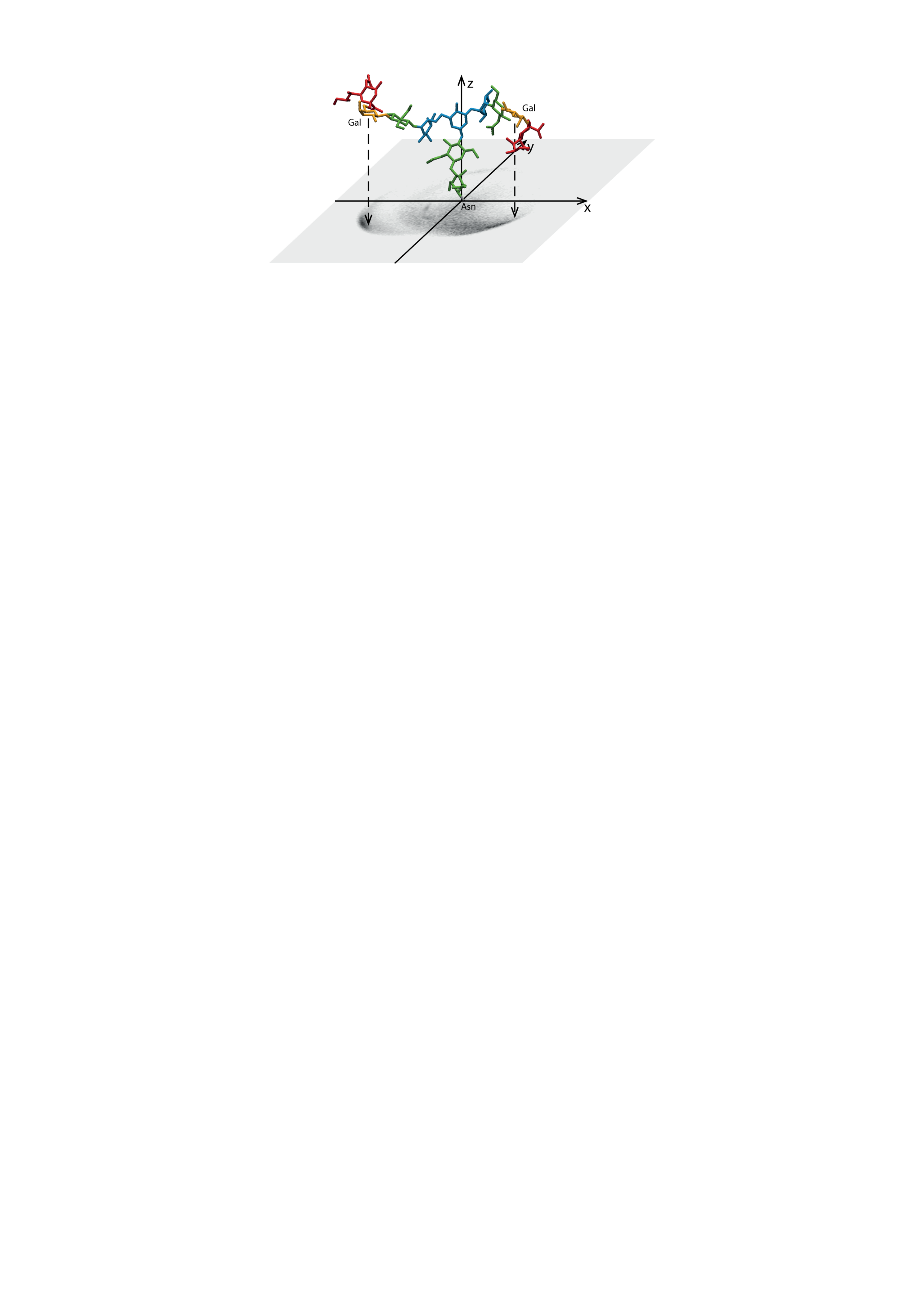
**

**Supplementary Figure S1.** “**Umbrella visualization” for a glycan chain.** The glycan chain is placed on a xyz coordinate system in such a way that the center of the asparagine residue is set on the origin and the inner-core is oriented along the z axis. At each time of the simulation, the position of the last block of each antenna is projected on the xy plan.

**
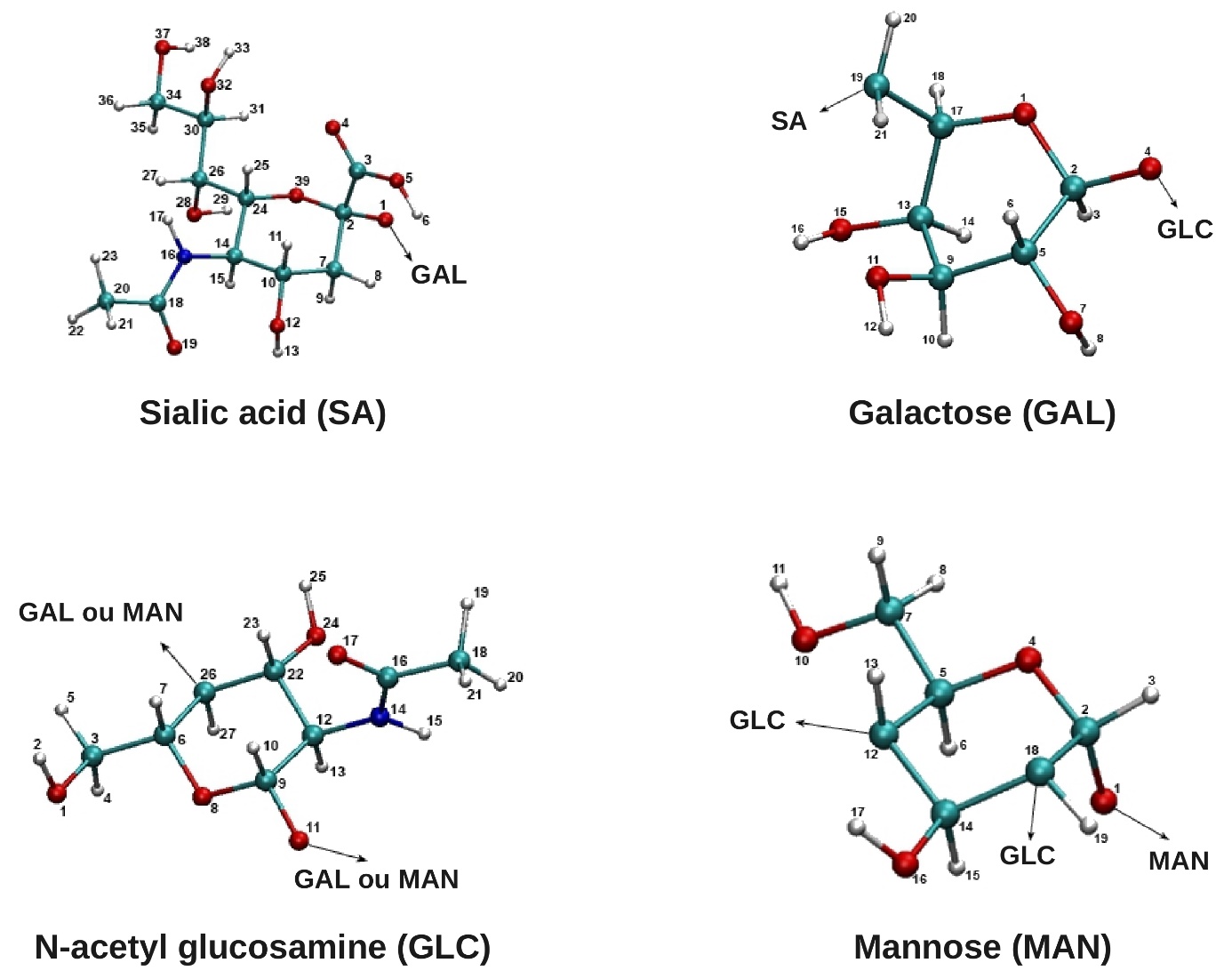
**

**Supplementary Figure S2.** **“Blocks” used in the description of the glycan chains.** In order to define the glycan chains five types of units were defined: Sialic acid (NeuAc or SIA), galactose (GAL), N acetylglucosamine (GlcNAc or GLC), Mannose (MAN) and Fucose (FUC). This figure presents the numbering of the atoms for the four units where parameters had to be adapted.
